# PPAR*α* Agonist WY-14643 Relieves Neuropathic Pain through SIRT1-Mediated Deacetylation of NF-*κ*B

**DOI:** 10.1155/2020/6661642

**Published:** 2020-12-14

**Authors:** Wanshun Wen, Jinlin Wang, Biyu Zhang, Jun Wang

**Affiliations:** ^1^Department of Rehabilitation Medicine, Zhejiang Provincial People's Hospital, People's Hospital of Hangzhou Medical College, Hangzhou, Zhejiang Province, China; ^2^Department of Anesthesiology, Wuhan Children's Hospital (Wuhan Maternal and Child Healthcare Hospital), Tongji Medical College, Huazhong University of Science & Technology, Wuhan, China; ^3^Department of Anesthesiology, Huai'an Second People's Hospital and the Affiliated Huai'an Hospital of Xuzhou Medical University, Huai'an, Jiangsu, China

## Abstract

Inflammation caused by neuropathy contributes to the development of neuropathic pain (NP), but the exact mechanism still needs to be understood. Peroxisome proliferator-activated receptor *α* (PPAR*α*), an important inflammation regulator, might participate in the inflammation in NP. To explore the role of PPAR*α* in NP, the effects of PPAR*α* agonist WY-14643 on chronic constriction injury (CCI) rats were evaluated. The results showed that WY-14643 stimulation could decrease inflammation and relieve neuropathic pain, which was relative with the activation of PPAR*α*. In addition, we also found that the SIRT1/NF-*κ*B pathway was involved in the WY-14643-induced anti-inflammation in NP, and activation of PPAR*α* increased SIRT1 expression, thus reducing the proinflammatory function of NF-*κ*B. These data suggested that WY-14643 might serve as an inflammation mediator, which may be a potential therapy option for NP.

## 1. Introduction

Neuropathic pain (NP), a complicated disease of the somatosensory nervous system, affects 7%-10% of the general population worldwide [1]. Patients who are diagnosed with NP will be accompanied with shooting and burning pain and tingling sensation so that their life quality decreases [2]. At present, the causative factors of NP is underestimated and the management of NP is on challenge [1]. Many studies show that there exists immune system dysfunction in NP, which leads to the process of allergic inflammation, as a way of the elevated proinflammatory cytokines and decreased anti-inflammatory cytokines [3–7]. However, the molecular mechanism of inflammation in NP still needs to be well established.

Peroxisome proliferator-activated receptor *α* (PPAR*α*) that belongs to PPAR families is a ligand-activated transcription factor that regulates lipid metabolism, neuronal survival, cardiac pathophysiology, cell cycle, and inflammation [8, 9]. It is reported that PPAR*α* exhibits inflammation-suppressing effects in obesity, atherosclerosis, autosomal dominant polycystic kidney disease, and acute kidney injury [10–13], but the role of PPAR*α* in inflammation in NP still remains unclear. WY-14643, as a PPAR*α* agonist, has been proved to reduce inflammation in several pathological processes [14]. Nevertheless, whether WY-14643 can suppress inflammation in NP is not elaborated.

Sirtuin 1 (SIRT1), a nicotinamide adenosine dinucleotide-dependent class III histone/protein deacetylase, participates in many cellular processes including aging, cell cycle, differentiation, apoptosis, metabolism, and inflammation [15, 16]. It is widely accepted that SIRT1 functioned as an inhibitor of nuclear factor-kappa B (NF-*κ*B) signaling and p65 acetylation was considered a pacemaker of the NF-*κ*B pathway [17]. Subsequent accumulating evidence shows that SIRT1 has the capacity of inhibiting inflammation by NF-*κ*B inhibition [18, 19].

In this study, we hypothesized that WY-14643 could alleviate the inflammation in NP via SIRT1/NF-*κ*B signaling. To verify the hypothesis, the chronic constriction injury (CCI) rat model was established. Pain tests were performed to examine whether WY-14643 could relieve the NP in CCI rats, and the expression of proinflammatory cytokines was detected to evaluate the inflammation in CCI rats.

## 2. Materials and Method

### 2.1. Animals

Adult male Sprague-Dawley rats (200-250 g, 8 weeks) were used in this study and purchased from Zhejiang Provincial People's Hospital. All rats were randomly divided into 10 groups (*n* = 6). The animal experiments were approved by People's Hospital of Hangzhou Medical College and performed in accordance with the *Guidelines for the Care and Use of Laboratory Animals* published by the National Institutes of Health (2011).

### 2.2. CCI Model

Chronic constriction injury (CCI) surgery was performed according to the procedure described by Bennett and Xie [20]. Rats were anesthetized with sodium pentobarbital (40 mg/kg, i.p.). An incision was made below the hip bone and parallel to the sciatic nerve. In the CCI rat groups, the bilateral sciatic nerves of two legs were exposed and ligated loosely by 4-0 chromic gut sutures with about 1 mm spacing, while nothing was ligated in the sham group. The surgery was performed by the same researcher. After operation, CCI rats gradually showed typical signs of spontaneous hyperalgesia, but the behavioral performance of the sham group is the same as before the operation.

After surgery, the CCI rats were treated with WY-14643 (10 mg/kg) [21]; GW6471 (30 mg/kg) [22]; and si-SIRT1 (250 nm/kg); the drugs were given by intrathecal injection. Two hours after the injection, pain tests were performed.

### 2.3. Pain Tests

#### 2.3.1. Mechanical Hyperalgesia

Paw withdrawal mechanical threshold (PWMT) [23] was determined applying electronic von Frey filament. Put the rats into plexiglass boxes (50 × 30 × 30 cm) with metal mesh floor. The filament was pressed on the plantar surface until the rats withdraw their paws. Record the values displayed by the electronic von Frey filament. Repeat each measurement 3 times at a 5-minute interval.

#### 2.3.2. Thermal Hyperalgesia

Paw withdrawal thermal latency (PWTL) [23] was determined using the thermal radiation. Put the rats into plexiglass boxes for more than 30 minutes to adapt to the environment. The heat source was pointed at the plantar surface of the hind paws, and the time when rats show paw withdrawal was recorded. The thermal stimulus was repeated 3 times at 5-minute interval, and the average value was considered PWTL. All behavioral tests were performed by the same person.

### 2.4. RNA Extraction and qRT-PCR

When all pain tests were finished, the rats were decapitated immediately. Collect the serum and store it in a -80°C refrigerator. The L4-6 spinal cord was frozen by liquid nitrogen. Total RNA of the frozen spinal cord was extracted using Trizol reagent (Invitrogen, USA), and the concentration of RNA was measured using NanoDrop 2000. Then, the reverse transcription PCR was performed using PrimeScript RT Master Mix (Takara, Japan). The qRT-PCR was performed using SYBR Premix Ex Taq (Takara, Japan) and run on the ABI 7500 Real-Time PCR System (Life Technologies, USA). GAPDH was used as internal control. The relative expression was calculated with the 2^-*ΔΔ*CT^ algorithm.

### 2.5. ELISA

The TNF-*α*, IL-1*β*, and IL-6 levels of serum and spinal cords were detected using Rat TNF-*α*, IL-1*β*, and IL-6 Precoated Kits (Dakewe, China).

### 2.6. Western Blot

The frozen samples were lysed using T-PER Tissue Protein Extraction Reagent (Thermo Scientific, USA). Protein concentration was measured by BCA Protein Assay Kit (Leagene, China). 30 *μ*g protein was separated by sodium dodecyl sulphate-polyacrylamide gel electrophoresis (SDS-PAGE) and transferred to polyvinylidene fluoride membranes. The membranes were incubated with primary antibodies (anti-SIRT1, 1 : 1000; anti-acetyl-NF-*κ*B p65, 1 : 1000) overnight at 4°C and secondary antibodies (1 : 5000) at room temperature for 1 h. Western ECL Substrate (Bio-Rad, USA) was used to visualize the protein bands. ImageJ was used to analyze the gray value of protein bands.

### 2.7. Statistical Analysis

Results were presented as the mean ± standard deviation. The significance of the difference between two groups is determined by Student's *t*-test; differences between more than three groups were analyzed by one-way analysis of variance. The SPSS 23.0 (IBM, USA) and GraphPad Prism 6. Ink (GraphPad, California) were used for major analysis. *P* < 0.05 was regarded statistically significant.

## 3. Results

### 3.1. Acute WY-14643 Treatment Temporarily Alleviates NP

**Figure 1:**
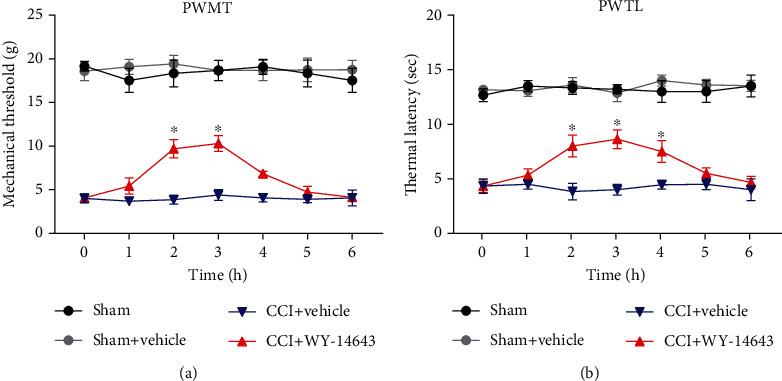
Acute WY-14643 treatment temporarily alleviated NP. (a) WY-14643 improved the paw withdrawal mechanical threshold (PWMT) of CCI rats. (b) WY-14643 improved the paw withdrawal thermal latency (PWTL) of CCI rats.

WY-14643 is a synthetic PPAR*α* agonist; current researches show that PPAR*α* plays a role in the development of NP. Therefore, to determine the antinociceptive effect of WY-14643 in NP, CCI rats were treated with WY-14643. Mechanical hyperalgesia and thermal allodynia were evaluated by PWMT and PWTL. Compared with the sham group, the PWMT and PWTL of the CCI group were significantly reduced. However, the PWMT was increased between 2 and 3 h after WY-14643 administration (Figure 1(a)). The PMTL was also improved between 2 and 4 h by WY-14643 treatment (Figure 1(b)). These results indicated that acute WY-14643 administration relieved NP temporarily.

### 3.2. Repeated WY-14643 Administration Alleviates NP in CCI Rats by Activating PPAR*α*

**Figure 2:**
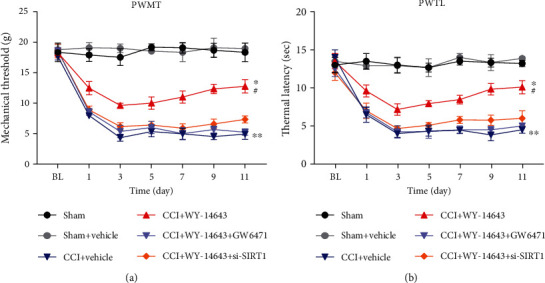
Repeated WY-14643 administration alleviated NP in CCI rats by activating PPAR*α*. (a) WY-14643 increased the PWMT of CCI rats, but GW6471 and si-SIRT1 reversed this effect. (b) WY-14643 increased the PWTL of CCI rats, but GW6471 and si-SIRT1 reversed this effect.

Given the above findings, we next tested whether repeated WY-14643 treatment could promote the recovery of NP in CCI rats. Rats were treated with WY-14643 once a day for 11 days after surgery. In addition, it has been reported that SIRT1 is involved in inflammation regulation and interacts with PPAR. Therefore, PPAR*α* antagonist GW6471 (MedChemExpress, USA) and in vivo si-SIRT1 (RiboBio, China) were also used to explore the mechanism of WY-14643 treating NP. Mechanical hyperalgesia was significantly reduced in the WY-14643 group, but GW6471 and si-SIRT1 aggravated the pain again (Figure 2(a)). Thermal hyperalgesia was also reduced by WY-14643 administration; however, GW6471 and si-SIRT1 reversed the antinociceptive effect of WY-14643 (Figure 2(b)). These data illustrated that WY-14643 relieved NP by acting as a PPAR*α* agonist, and SIRT1 inhibition blocked the function of WY-14643 in NP.

### 3.3. Repeated WY-14643 Administration Reduces Inflammation in CCI Rats by Activating PPAR*α*

**Figure 3:**
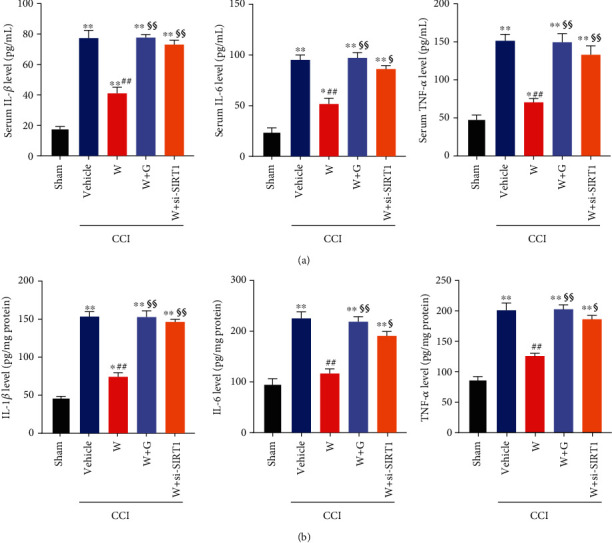
Repeated WY-14643 administration reduced inflammation in CCI rats by activating PPAR*α*. (a) WY-14643 reduced IL-1*β*, IL-6, and TNF-*α* levels in serum of CCI rats, whereas GW6471 and si-SIRT1 reversed the anti-inflammatory effect. (b) WY-14643 reduced IL-1*β*, IL-6, and TNF-*α* levels in the spinal cord of CCI rats, whereas GW6471 and si-SIRT1 reversed the anti-inflammatory effect.

Recent studies indicate that activation of PPAR*α* increases the expression and activity of SIRT1, which leads to deacetylation of p65 NF-*κ*B, thus inhibiting the expression of inflammatory cytokines [24, 25]. Since inflammation has been reported to be involved in the pathogenesis of neuropathic pain, the expression of proinflammatory cytokines including IL-1*β*, IL-6, and TNF-*α* was determined using ELISA. The serum and spinal cord samples were collected immediately after finishing all behavior tests. In CCI rats, IL-1*β*, IL-6, and TNF-*α* levels in serum were significantly increased (Figure 3(a)). Repeated WY-14643 treatment decreases the levels of these proinflammatory cytokines. However, PPAR*α* antagonist (GW6471) and si-SIRT1 increase IL-1*β*, IL-6, and TNF-*α* levels in serum (Figure 3(a)). We also detected the expression of IL-1*β*, IL-6, and TNF-*α* in the spinal cord. WY-14643 markedly suppressed the inflammation in CCI rats, whereas GW6471 and si-SIRT1 reversed the anti-inflammatory effect induced by WY-14643 (Figure 3(b)). Taken together, the results showed that WY-14643 reduced the inflammation in NP through the PPAR*α*/SIRT1 pathway.

### 3.4. SIRT1/NF-*κ*B Pathway Mediates WY-14643-Induced Inhibition of NP

**Figure 4:**
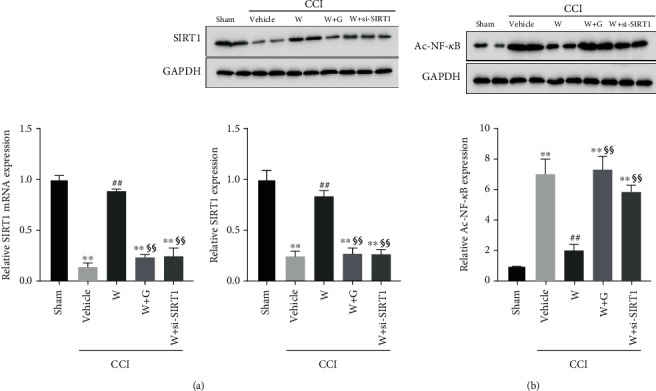
WY-14643 mediated the SIRT1/NF-*κ*B pathway by activating PPAR*α*. (a) The expression of SIRT1 in different groups was detected by qRT-PCR and western blot. WY-14643 administration increased SIRT1 expression. (b) The expression of acetylated NF-*κ*B p65 in different groups was detected by western blot. WY-14643 administration decreased acetylated NF-*κ*B p65 expression, but SIRT1 inhibition increased acetylated NF-*κ*B p65 expression. The relative protein expression was normalized according to the gray value of the band, which was analyzed by ImageJ.

NF-*κ*B is a key inflammatory mediator, which participates in the regulation of inflammatory response. SIRT1, a nicotinamide adenine dinucleotide-dependent deacetylase, has been shown to inhibit NF-*κ*B signaling by deacetylating the p65 subunit of NF-*κ*B complex [24]. To further explore the molecular mechanism of NP, we detected the expression of SIRT1 and acetylated NF-*κ*B p65 (Ac-NF-*κ*B p65). CCI decreases SIRT1 expression (Figure 4(a)), whereas Ac-NF-*κ*B p65 expression was significantly increased (Figure 4(b)). WY-14643 treatment increased SIRT1 expression, thus deacetylating NF-*κ*B p65. But GW6471 and si-SIRT1 inhibited the expression of SIRT1 and restored the Ac-NF-*κ*B p65 expression (Figures 4(a) and 4(b)). These results demonstrated that WY-14643 alleviated NP through the PPAR*α*-mediated SIRT1/NF-*κ*B pathway in CCI rats.

## 4. Discussion

The underlying basis of NP is the chronic ectopic electrical activity of nociceptive neurons. The cells located in the injury site release proinflammatory cytokines, leading to a proinflammatory environment [7]. Therefore, a deep understanding of how to reduce inflammation is urgent for NP treatment. PPAR*α* has gained great attention for its anti-inflammatory effects in many disease models. However, the molecular mechanism between PPAR*α* activation and inflammation in CCI model has not been fully elucidated. In this study, we first used PPAR*α* agonist WY-14643 to explore the signaling pathway between the PPAR*α* activation and inflammation in NP.

Firstly, we found that the PWMT and PWTL of CCI rats were significantly lower than those of the sham group, whereas PPAR*α* agonist WY-14643 treatment would increase the PWMT and PWTL. Subsequently, we compared the effects of WY-14643 and PPAR*α* antagonist GW6471 on the CCI rats. The significant reduction of mechanical and thermal hyperalgesia in WY-14643-treated group was observed; however, GW6471 administration blocked these effects. The above results suggested that WY-14643 owned potential treatment value for NP. And this is the first time that the function of WY-14643 in NP was studied.

Previous evidences demonstrated that PPAR*α* could activate SIRT1 and play a role in many biological processes. Oka et al. reported that the PPAR*α*/SIRT1 pathway took part in the progression of heart failure by promoting mitochondrial dysfunction [26]. Ogawa et al. found that the repression of microglial activation was associated with SIRT1-dependent PPAR*α* signaling [27]. Sandoval-Rodriguez et al. showed that PPAR*α* improved nonalcoholic steatohepatitis via acting on SIRT1 [28]. However, whether the PPAR*α*/SIRT1 pathway was involved in NP development was unclear. In CCI rats, we found that mechanical and thermal hyperalgesia was downregulated in the WY-14643 group, whereas coadministration of si-SIRT1 and WY-14643 would recover the damage again in rats. These findings proved that the PPAR*α*/SIRT1 pathway might participate in the pathogenesis of NP.

**Figure 5:**
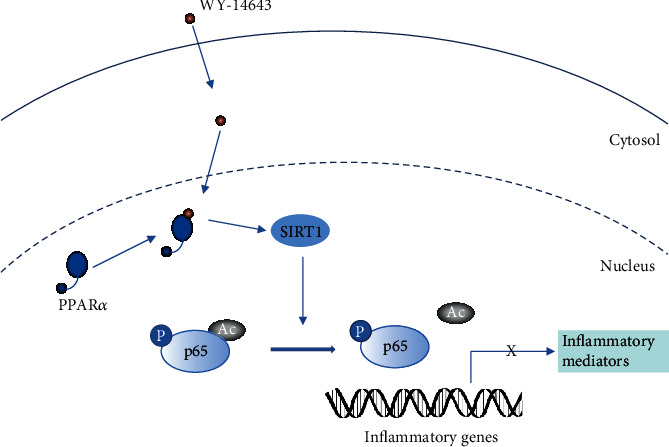
The PPAR*α*/SIRT1/NF-*κ*B pathway in the inflammation in NP.

To explore the effects of PPAR*α*/SIRT1 signaling on inflammation, we detected the expression of inflammatory cytokines and noticed that the inflammatory cytokines were evidently increased in CCI rats, but WY-14643 treatment restrained the inflammation. Further treatment with si-SIRT1 would reverse the anti-inflammatory effect stimulated by WY-14643. It suggested that PPAR*α*/SIRT1 signaling might be relative to the inflammation progression of NP. The results were consistent with the clues that PPAR*α* had a hand in inflammation by regulating SIRT1 [28]. Moreover, since SIRT1 was capable of bating inflammation by inhibition of NF-*κ*B [15, 17], we also determined the levels of SIRT1 and acetylated NF-*κ*B p65 in CCI rats. WY-14643 could make SIRT1 activated and deacetylate NF-*κ*B p65, but GW6471 and si-SIRT1 would reduce the expression of SIRT1 and upregulate the expression of Ac-NF-*κ*B p65. The regulating pathway is shown in Figure 5.

However, there are still limitations in our study. Currently, sufficient evidence to support the clinical application of WY-14643 for NP is lacking, and the side effects of WY-14643 are uncertain. Consequently, the safety of WY-14643 should be further considered. And we will also further explore the safety of WY-14643 in the future.

In conclusion, our study illustrates that PPAR*α* agonist WY-14643 relieves inflammation in NP via the SIRT1/NF-*κ*B pathway.

## Data Availability

All data are available upon request.
